# A211 CLINICAL CHARACTERISTICS OF INFLAMMATORY BOWEL DISEASE IN JORDANIAN CHILDREN: A RETROSPECTIVE STUDY

**DOI:** 10.1093/jcag/gwae059.211

**Published:** 2025-02-10

**Authors:** E Altamimi, A S Alsmadi, H H Almomani, M A Alshurman

**Affiliations:** Pediatrics and Neonatology, Jordan University of Science and Technology, Irbid, Irbid, Jordan; Pediatrics and Neonatology, Jordan University of Science and Technology, Irbid, Irbid, Jordan; Pediatrics and Neonatology, Jordan University of Science and Technology, Irbid, Irbid, Jordan; Jordan University of Science and Technology, Irbid, Jordan

## Abstract

**Background:**

Inflammatory Bowel Disease (IBD) is a chronic condition characterized by inflammation of the gastrointestinal tract. While IBD is increasingly recognized in pediatric populations worldwide, there is limited data on its characteristics in Jordanian children.

**Aims:**

This study aims to describe the clinical presentation and management outcomes of IBD in children from a tertiery center in North Jordan.

**Methods:**

A retrospective analysis was conducted on pediatric patients (aged 0-18 years) diagnosed with IBD between July 2017 and July 2024 at King Abdullah University Hospital in North Jordan. Data on demographic information, clinical symptoms, diagnostic procedures, treatment regimens, and follow-up outcomes were collected from medical records.

**Results:**

Over the study period, 31 patients were diagnosed with IBD, with 58% being female. Among these, 51.6% were classified as Crohn’s disease, 29.0% as ulcerative colitis, and 19.4% as indeterminate colitis. The mean age at diagnosis was 8.7 years. The most common presenting symptoms were diarrhea (67.6%), abdominal pain (58.0%), weight loss (32.2%), and fatigue/malaise (32.2%).

Anemia was observed in 17 patients (54.8%). Intravenous iron therapy was required in 3 patients (9.7%), while 2 patients (6.5%) needed multiple blood transfusions. Escalation of initial therapy was necessary for 15 patients (48.4%). Biologic therapy (Anti-TNF agents) was used in 8 patients (25.8%), all of whom initially responded. However, 3 patients (37.5%) were switched to a second anti-TNF agent after secondary loss of response. Two patients (25.0%) developed psoriatic lesions, although none experienced serious infections. Notably, one patient (12.5%) required concomitant anti-tuberculosis treatment due to a latent tuberculosis diagnosis prior to initiating biologics.

One patient (3.2%) developed pancreatitis requiring hospitalization. This same patient also experienced dural venous thrombosis, necessitating prolonged anticoagulant therapy. None of the patients in this cohort underwent surgical intervention.

**Conclusions:**

IBD in Jordanian children presents with clinical features similar to those reported in other regions, although environmental or genetic factors may lead to some variations in disease patterns, and treatments` response. Further studies are needed to optimize management protocols for pediatric IBD in Jordan.

**Funding Agencies:**

None

